# The anti-NGF antibody muMab 911 both prevents and reverses pain behaviour and subchondral osteoclast numbers in a rat model of osteoarthritis pain

**DOI:** 10.1016/j.joca.2016.05.015

**Published:** 2016-09

**Authors:** L. Xu, L.N. Nwosu, J.J. Burston, P.J. Millns, D.R. Sagar, P.I. Mapp, P. Meesawatsom, L. Li, A.J. Bennett, D.A. Walsh, V. Chapman

**Affiliations:** † Arthritis Research UK Pain Centre, University of Nottingham, Nottingham, UK; ‡ School of Medicine, University of Nottingham, Clinical Sciences Building, City Hospital, Hucknall Road, Nottingham, NG5 1PB, UK; § School of Life Sciences, University of Nottingham, Queen's Medical Centre, Nottingham, NG7 2UH, UK

**Keywords:** Osteoarthritis, Pain, Bone, Inflammation, Nerve growth factor

## Abstract

**Objective:**

Nerve growth factor (NGF) has a pivotal role in peripheral hyperalgesia and inflammation; anti-NGF antibodies attenuate pain responses in inflammatory pain models, and in people with osteoarthritis (OA) or low back pain. The aim of this study was to characterise the peripheral mechanisms contributing to the analgesic effects of anti-NGF antibody treatment in an established model of joint pain, which mimics key clinical features of OA.

**Design:**

Effects of preventative vs therapeutic treatment with an anti-NGF antibody (monoclonal antibody 911: muMab 911 (10 mg/kg, s.c.)) on pain behaviour (weight bearing asymmetry and hindpaw withdrawal thresholds (PWT)), cartilage damage, synovitis and numbers of subchondral osteoclasts were investigated in the monosodium iodoacetate (MIA) model. Potential direct effects of NGF on receptor activator of nuclear factor kappa-B ligand (RANKL) mediated osteoclastogenesis were investigated in cultured human osteoclasts.

**Results:**

Intra-articular MIA injection resulted in significant pain behaviour, cartilage damage, synovitis and increased numbers of subchondral osteoclasts. Both preventative and therapeutic treatment with muMab 911 significantly prevented, or reversed, MIA-induced pain behaviour, but did not alter cartilage or synovial pathology quantified at the end of the treatment period. NGF did not facilitate RANKL driven osteoclast differentiation *in vitro*, but preventative or therapeutic muMab 911 reduced numbers of TRAP positive osteoclasts in the subchondral bone.

**Conclusions:**

We demonstrate that anti-NGF antibody treatment attenuates OA pain behaviour despite permitting cartilage damage and synovitis. Indirect effects on subchondral bone remodelling may contribute to the analgesic effects of NGF blockade.

## Introduction

Osteoarthritis (OA) is the most common form of arthritis and the fastest growing chronic pain disease worldwide^1^, prevalence of OA increases with age^2^. All compartments of OA joints undergo structural changes, which impacts upon function, causing disability and reducing quality of life^2^. Joint structural changes associated with OA pain include synovitis^3^ and bone marrow lesions^4^. OA pain is characterised by both pain on loading and spread of pain to sites remote from the diseased joint, and clinical evidence supports a contribution of both peripheral and central sensitization mechanisms to OA pain^5^. The challenge of identifying analgesic strategies that more effectively target OA pain requires a comprehensive understanding of the underlying pain mechanisms. Rodent models which mimic aspects of the clinical pathology and exhibit pain on loading (weight bearing) and lowering of paw withdrawal thresholds (PWT) at remote sites^6^ play a pivotal role in elucidating mechanisms of OA pain, which cannot be readily interrogated in clinical studies.

Nerve growth factor (NGF) is widely recognised as a mediator of chronic pain. NGF binds to high affinity tropomyosin-receptor-kinase A (TrkA) and low affinity p75 neurotrophin receptors on sensory nerve terminals and immune cells, and has an established role in peripheral hyperalgesia associated with inflammation^7^. Injection of NGF into the knee induces pain behaviour and synovitis in naïve rats, and exacerbates established pain behaviour in a model of OA pain^8^. The neuronal basis for these behavioural effects of exogenous NGF is likely to include an expansion of knee joint peripheral receptive fields of spinal neurones^9^. NGF levels are increased in arthritic joints, within the inflamed synovium and osteochondral junction^10^, ^11^. NGF is known to sensitise peripheral nerves and might stimulate blood vessel and nerve growth into structures such as the articular cartilage that are not normally innervated^12^. However, whether endogenous NGF contributes to the synovitis and joint pathology associated with OA pain, as well as the direct sensitization of sensory nerves, is unknown. NGF blockade can be achieved using antibodies or TrkA-IgG fusion protein that bind NGF and prevent its interaction with TrkA and p75 receptors. Humanised monoclonal antibodies against NGF have robust analgesic effects in OA sufferers^13^, ^14^. However, unexpected findings of rapidly progressive OA (RPOA) in some participants, unrelated to the extent of pain relief^15^, has highlighted the need for further mechanistic studies. To address this gap in knowledge the aim of the present study was to investigate the peripheral mechanisms that underpin the analgesic effects of sequestering NGF. To this end we have compared the effects of preventative vs therapeutic treatment with the anti-NGF sequestering antibody muMab 911 on two indices of pain, weight bearing asymmetry (pain on loading) and lowered hindpaw withdrawal thresholds (distal pain) with parallel measures of joint pathology (chondropathy, synovitis and numbers of subchondral osteoclasts) in an established model of OA pain. To determine whether a potential direct effect of NGF on osteoclast differentiation underpins the inhibitory effects of muMab 911 *in vivo*, effects of NGF treatment on RANKL and non-RANKL mediated differentiation of human monocytes into osteoclasts was studied.

## Methods

### Animal and model induction

Studies were conducted in accordance with UK Home Office Animals (Scientific Procedures) Act (1986) and guidelines of the International Association for the Study of Pain. Study approval was granted by the University of Nottingham Ethics committee. The experimenter was blind to all interventions. 130 male Sprague Dawley rats (140–260 g (5–7 weeks old) Charles River UK) were group housed, had free access to food and water and were on a 12 h light/dark cycle. Group sizes were *n* = 10, data points which were statistical outliers (median ± 2 standard deviations) were excluded and final numbers are reported below. Rats were anaesthetised (isoflurane) and received intra-articular injection of monosodium iodoacetate (MIA; 1 mg/50 μl; Sigma UK) in saline, or saline (50 μl) as a control, through the infra-patellar ligament of the left knee on Day 0^6^. Post-injection rats were group housed (5 per cage) and their posture and behaviour was closely monitored for 48 h. Pain behaviour was assessed in the laboratory in the morning prior to intra-articular injection on Day 0 as weight bearing asymmetry (using an incapacitance tester (Linton Instrumentation, UK), a behavioural correlate of hyperalgesia, and hind paw mechanical withdrawal thresholds (Semmes-Weinstein monofilaments of bending forces 1–26 g), an index of central sensitization^6^. Four independent studies were performed in MIA and saline control rats: effect of preventative treatment vs vehicle on pain behaviour and joint pathology up to day 28 (data reported on 38 rats); effect of therapeutic treatment vs vehicle on pain behaviour and joint pathology up to day 28 (data reported on 38 rats); effect of a preventative treatment of IgG1 vs vehicle on pain behaviour up to day 28 (data reported on 30 rats) evaluation of joint pathology at day 14 in MIA and saline control rats (data reported for 19 rats).

### Drug treatments and administration protocols

Rats were habituated to the pain behavioural tests and subcutaneous injections. Effects of the anti-NGF sequestering antibody (monoclonal antibody 911, muMab 911; provided by Dr David Shelton, Rinat/Pfizer, San Francisco, CA)^16^ were determined in the MIA model. MuMab 911 blocks binding of NGF to TrkA and p75^NTR^ receptors, inhibits TrkA auto-phosphorylation^16^, has a plasma half-life of 5–6 days in the rodent and low blood brain barrier permeability^17^. The preventative study used weekly dosing of muMab 911 (s.c. 10 mg/kg) or PBS from Day 0 (Supplementary fig. 1(A), *n* = 8–10 rats per group). Two types of control were used, magnesium and calcium free phosphate buffered saline (PBS) as the vehicle control and an IgG control specific for muMab 911 (s.c. 10 mg/kg), which was administered to a separate cohort of rats using the preventative treatment protocol identical to that outlined in Supplementary fig. 1(A) (*n* = 10 rats per group). The therapeutic study used dosing of muMab 911 (s.c. 10 mg/kg) or PBS on Day 14 and 21 post-MIA injection (Supplementary fig. 1(B)). MIA groups were matched according to weight bearing asymmetry prior to treatment by an independent experimenter. Cohorts consisted of both sham and MIA rats treated with either vehicle or muMab 911, experimenters blinded to all treatments. This dose of muMab 911 attenuated hyperalgesia in auto-immune arthritis^17^ and bone cancer pain^18^. Pain behaviour was assessed twice weekly after model induction, studies were terminated on Day 28 (Supplementary fig. 1). On the basis of previous unpublished studies, muMab 911 was expected to cause a minor skin irritation in some rats. These were quantified as mild if the skin lesion was <0.4 cm in diameter without bleeding, moderate if there was one or more area(s) of skin lesion with a diameter >0.4 cm, with or without bleeding. Rats exhibiting either mild or moderate skin irritation were treated topically with fuciderm gel (0.5% w/w Fusidic acid, 0.1% w/w Betamethasone).

### Histology

Rats were overdosed with pentobarbital (i.p.) at sacrifice. Tibiofemoral joints were removed and post-fixed in neutral buffered formalin (4% formaldehyde), decalcified in 10% ethylenediaminotetraacetic acid (EDTA) for 6–8 weeks^6^ and embedded in paraffin wax. Histomorphometry was carried out by an observer blinded to treatment. Full details of the tissue section preparation and processing is provided in the supplementary section. Coronal tissue sections were cut at 5 μm following the Osteoarthritis Research Society International (OARSI) guideline for histological assessment for OA in the rat^19^. Haematoxylin and eosin stained sections were scored for articular cartilage surface integrity and synovial inflammation^6^, ^20^. Quantification of numbers of tartrate-resistant acid phosphatase (TRAP) positive osteoclasts was perform as previously described^21^.

### *In vitro* model of human osteoclast differentiation

This study was approved by the Nottingham University Medical School Research Ethics Committee. Full details of the methods of *in vitro* model of human osteoclast differentiation is provided in the Supplementary section. In brief, peripheral blood from healthy human donors was collected and blood monocytes were isolated from buffy coats by gradient centrifugation, monocytes were seeded onto glass coverslips within a 24-well culture plates, and cultured in growth media supplemented with human macrophage colony stimulating factor (MCSF; R&D Systems) and with 30 ng ml^−1^ of human RANKL (Santa Cruz), unless otherwise stated. Cells were incubated at 37°C, 7% CO^2^ for 2 h, and the medium replaced. Growth media containing NGF (0–200ngml^−1^ was then added to the cells. After 14 days, cells were washed with Hanks buffered saline solution, fixed with 10% neutral buffered formalin, washed and stored at 4°C in PBS containing 0.01% w/v sodium azide.

Differentiated osteoclasts were identified by TRAP staining using the commercial kit described above. For quantification of TRAP positive cells four random fields of view were counted per coverslip using four coverslips per condition. Cells that stained positive for TRAP and had three or more nuclei were counted.

### Statistical analysis

Data were tested for normality prior to statistical analysis. Data points were classified as outliers if they exceeded the mean ± 2 standard deviations, final group sizes are reported in figure legends. Comparisons of pain behaviour between groups of rats at different time points were carried out using two-way ANOVA with Bonferroni's *post-hoc* tests. To address potential multiplicity, area under the curve analysis of timecourse data was also performed with a Mann–Whitney *U*-test. Comparisons of osteoclast numbers between groups used a one-way ANOVA with Bonferroni's *post-hoc* test. Associations between osteoclast numbers and pain behaviour in MIA-injected rats treated with muMab 911 or vehicle were tested by pooling data from preventative and therapeutic treatment protocols, using linear (weight bearing asymmetry) or logistic (PWT) regression, with adjustment for possible between experiment variation by including experiment number as a covariate. Due to the ordinal nature of joint structure and inflammation scores, data were non-normally distributed and comparisons between groups used a Kruskal Wallis test with post hoc Dunn's test. *P* < 0.05 was statistically significant. Analysis of *in vitro* TRAP assays was performed with a either a one way ANOVA with Dunnett's test (more than two group compared) or with a unpaired two-tailed Mann–Whitney *t*-test (two groups compared).

## Results

### Effects of preventative muMab 911 on MIA-induced pain behaviour and changes in joint structure

**Fig. 1:**
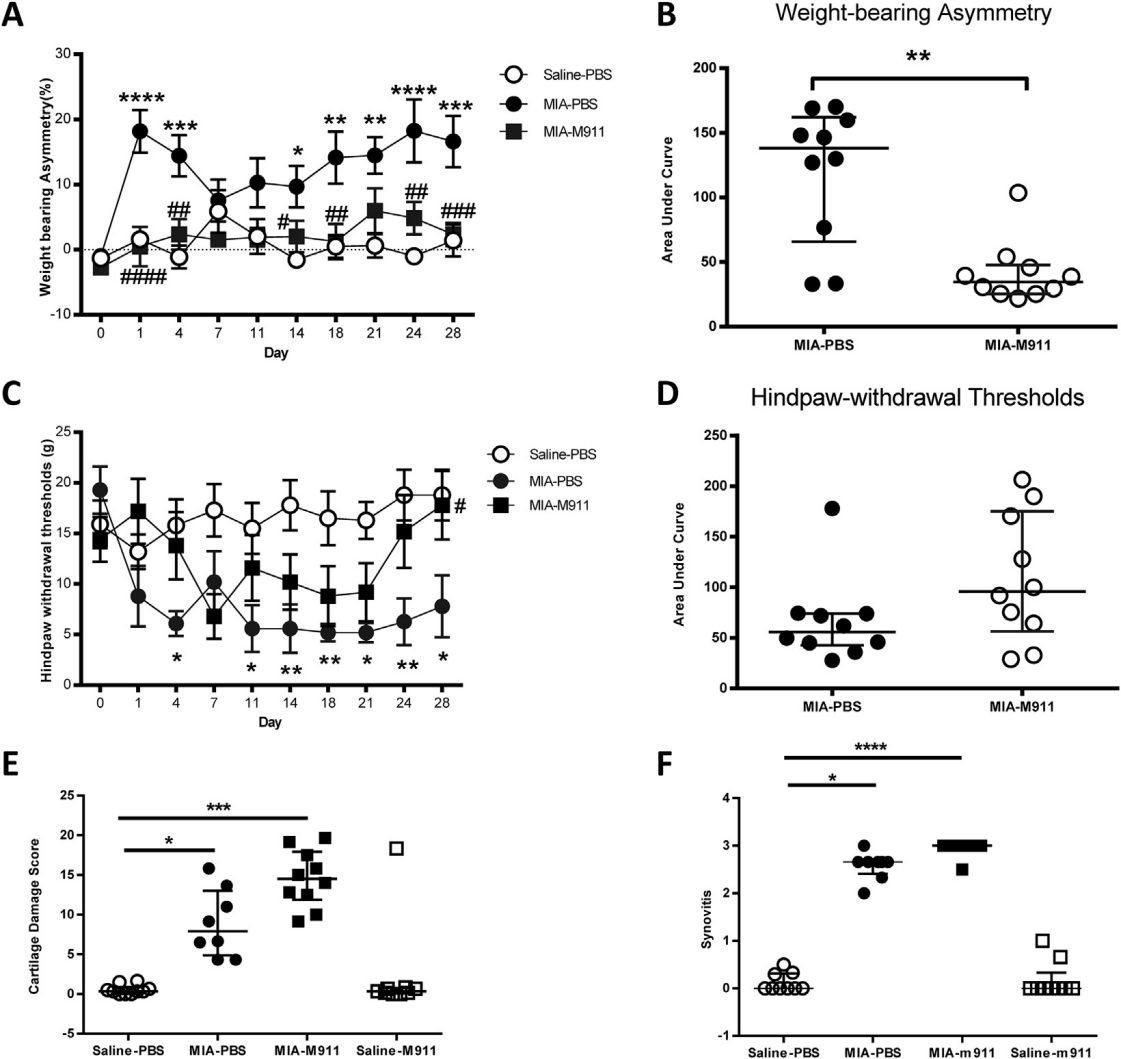
**Preventative muMab 911 attenuates OA pain behaviour but not cartilage damage or synovitis in the MIA model of OA pain**. Rats received weekly subcutaneous injection of 10 mg/kg muMab 911 or PBS on days 0, 7, 14, and 21 post intra-articular injection of MIA or saline. Preventative muMab 911 robustly prevented MIA-induced changes in weight-bearing asymmetry (A, B) and attenuated hindpaw withdrawal thresholds (C, D). Statistical comparison of groups at each timepoint: two-Way ANOVA with Bonferroni's *post-hoc* tests, *P < 0.05, **P < 0.01, ***P < 0.001: MIA vs saline; #*P* < 0.05, ##*P* < 0.01, ###*P* < 0.001 muMab 911 vs PBS. Note saline muMab 911 group did not differ from the saline PBS group, and is not shown for clarity (*n* = 10 rats per group). Preventative treatment with muMab 911 did not significantly alter MIA-induced cartilage damage (*n* = 9–10 per group) (E) or synovial inflammation (*n* = 8–9 per group) (F). *P < 0.05, **P < 0.01, ***P < 0.001 vs saline-PBS. Comparisons between Areas Under Curve were performed using a Mann–Whitney *U*-test. Comparisons of histology between groups used a Kruskal Wallis test with Dunn's post hoc. A, C: data are mean ± SEM; B, D, E, F: data are median and interquartile range. M911: muMab 911.

Intra-articular injection of MIA was associated with a significant increase in weight bearing asymmetry and a significant decrease in ipsilateral hindpaw withdrawal thresholds, compared to intra-articular injection of saline [Fig. 1(A), (C)]. Preventative treatment with muMab 911 (10 mg/kg weekly) from Day 0 robustly prevented the development of weight-bearing asymmetry (Fig. 1(A); F(3,360 = 40.88, MS = 2816, *P* < 0.0001). Effects of muMab 911 on MIA-induced lowering of hindpaw withdrawal thresholds were less immediate, and only significant on day 28 [Fig. 1(C)]. AUC analysis only revealed a significant effect of muMab 911 on weight-bearing asymmetry [Fig. 1(B), (D)]. MuMab 911 did not alter weight-bearing or hindpaw withdrawal thresholds in saline injected rats (data not shown). Preventative administration of control IgG1 did not alter MIA-induced changes in weight bearing or PWT, compared to PBS-treated MIA-injected rats (Supplementary fig. 2).

**Fig. 2:**
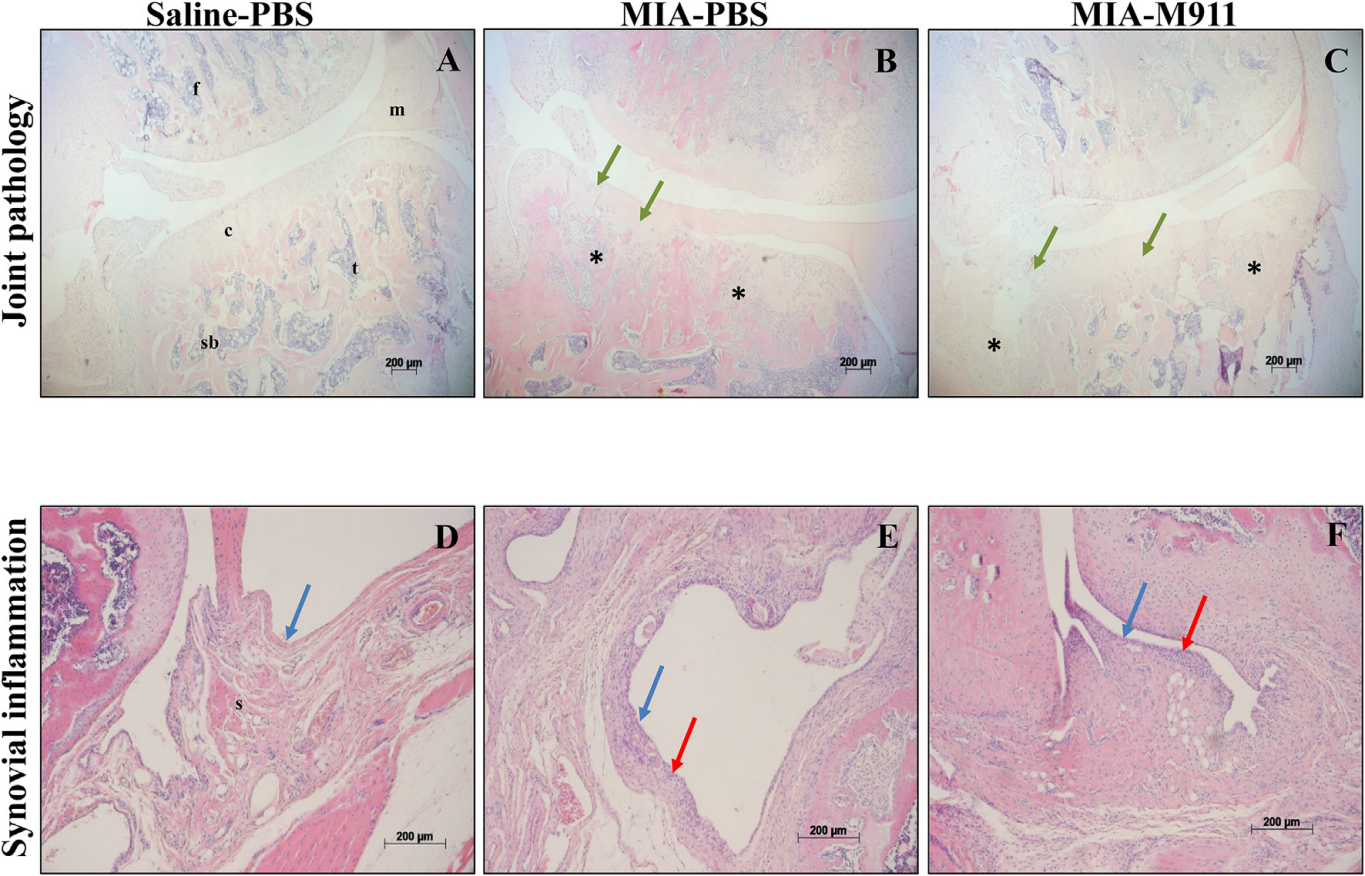
**Preventative muMab 911 and cartilage damage or synovitis in the MIA model of OA pain**. Histological sections of osteochondral (A–C) or synovial (D–F) tissue. **A**; PBS-treated saline-injected control showing an intact joint with smooth cartilage, normal joint margin and chondrocyte morphology. **B**; 1 mg MIA-injected PBS-treated rat showing OA structural pathology: cartilage damage (green arrows); chondrocyte loss (green arrows); subchondral bone changes (asterisk). **C**: M911-treated MIA-injected rat exhibited similar pathology to the MIA-PBS treated rat (B). **D**: 1–2 cell deep synovial lining layer (blue arrow) in PBS-treated saline-injected control. **E**; Synovial hyperplasia (red arrows) in 1 mg MIA-injected PBS-treated rat. **F**: Synovium from a M911-treated MIA-injected rat exhibited similar pathology to the MIA-PBS treated rat (E). Photomicrographs show haematoxylin and eosin stained sections of knee tissue from a rat with the median pathology score from each group. Scale bars = 200 μm. F = femur, m = meniscus, c = cartilage, t = tibia, sb = subchondral bone and s = synovium.

The MIA model was associated with changes in the articular cartilage of the knee joint, measured as joint damage score [Fig. 1(E)] and synovial inflammation [Fig. 1(F)], compared to saline injected rats [Fig. 2(A)–(F)]. Preventative treatment with muMab 911 did not significantly alter these MIA-induced changes in the knee joint [Fig. 1(E), (F), Fig. 2(A)–(F)].

**Fig. 3:**
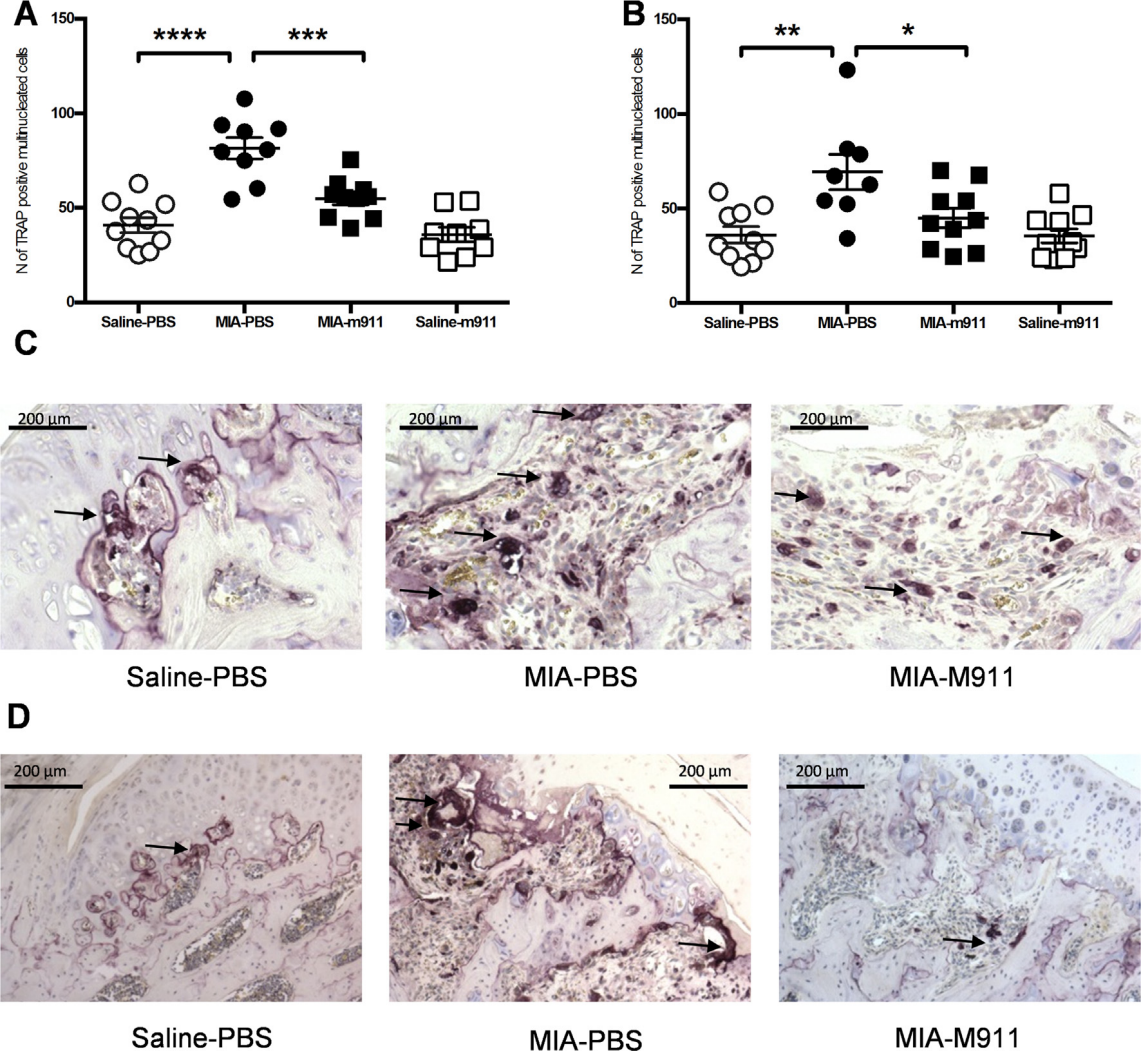
**Preventative and therapeutic muMab 911 attenuates the number of TRAP positive osteoclasts in the tibial plateau in MIA rats**. (A) The number of TRAP positive osteoclasts in the tibial plateau was significantly increased in MIA rats receiving vehicle treatment. Preventative treatment with muMab 911 significantly reduced the number of TRAP positive osteoclasts in MIA rats. Data are mean ± SEM, *n* = 9–10 per group. (B) Therapeutic muMab 911 significantly attenuated the MIA-induced increase in the number of TRAP positive osteoclasts in the tibial plateau. Data are mean ± SEM *n* = 8–10 per group. Statistical comparison was carried out using one-Way ANOVA with Bonferroni's *post-hoc* tests, *P < 0.05, **P < 0.01. Representative images of osteoclasts at the osteochondral junction of the tibial plateau in a saline-PBS rat (left), MIA-PBS rat (middle) and MIA-muMab 911 rat (right) in the preventative study (C) and therapeutic study (D). Images were taken with a Zeiss Axioplan microscope at 10× magnification. Arrows indicate multinucleated TRAP positive osteoclasts. M911: muMab 911.

The MIA model was also associated with an increase in the number of TRAP positive osteoclasts at the tibial plateau, compared to saline injected rats [Fig. 3(A), (C)]. There was a significantly higher number of TRAP positive osteoclasts at the tibial plateau of MIA rats receiving preventative vehicle, compared to MIA rats receiving preventative muMab 911 (Fig. 3(A); F (3, 34) = 22.83, MS = 3838, *P* < 0.0001). There was no significant difference in the number of TRAP positive osteoclasts at the tibial plateau from MIA-injected rats treated with preventative muMab 911 and saline injected rats [Fig. 3(A), (C)].

### Effects of therapeutic muMab 911 on MIA-induced pain behaviour and changes in joint structure

**Fig. 4:**
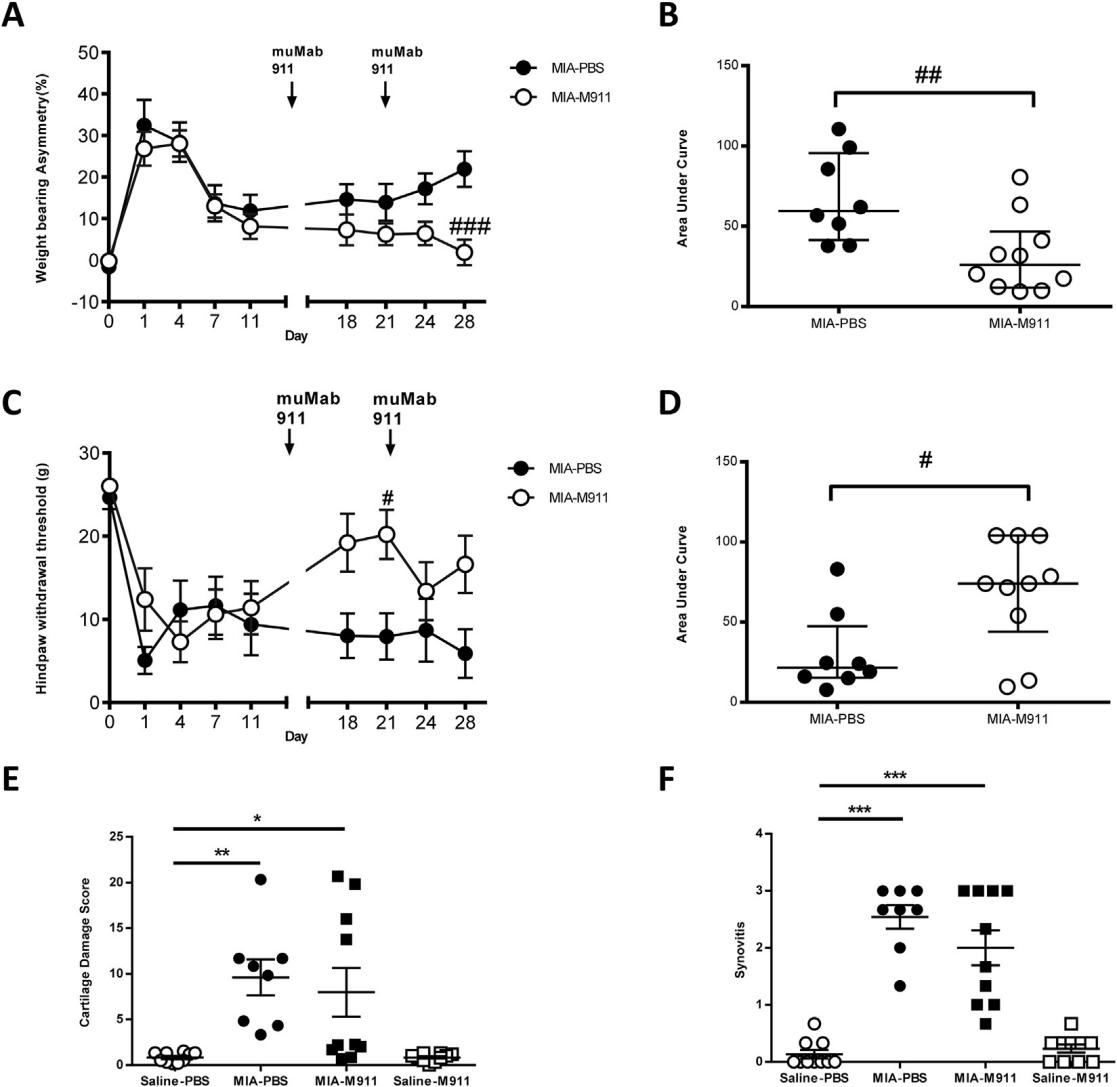
**Therapeutic muMab 911 attenuates established OA pain behaviour but not cartilage damage and synovitis in the MIA model of OA pain**. Rats received subcutaneous injection of 10 mg/kg muMab 911 or PBS on days 14 and 21 post intra-articular injection of MIA. muMab 911 treatment alleviated MIA-induced changes in weight-bearing asymmetry (A, B) and attenuated hindpaw withdrawal thresholds (C, D). Statistical comparison of muMab 911 and PBS treatment on pain behaviour, A, C are mean ± SEM (*n* = 8–10 rats per group): two-Way ANOVA with Bonferroni's *post-hoc* tests, #*P* < 0.05, ###*P* < 0.001. Note behavioural data from rats which received intra-articular injection of saline were comparable to the preventative study, and are not shown for clarity. B, D are median and interquartile range, comparison of these data was performed with a Mann–Whitney *U*-test, #*P* < 0.05, ##*P* < 0.01. Therapeutic treatment with muMab 911 did not significantly alter MIA-induced cartilage damage (E) or synovial inflammation (F) (*n* = 8–10 rats per group). E, F are median and interquartile range, comparisons between groups used a Kruskal Wallis test with Dunn's post hoc comparison. *P < 0.05, **P < 0.01, ***P < 0.001. M911: muMab 911.

The MIA model was associated with a significant increase in weight bearing asymmetry and lowering of hindpaw withdrawal thresholds, there were no differences between the magnitudes of these measures in the two groups of MIA rats prior to the start of the therapeutic treatment [Fig. 4(A), (C)]. Therapeutic muMab 911 (10 mg/kg, s.c, day 14 and 21) significantly reversed pain behaviour in MIA-injected rats (Fig. 4(A); F(3,340) = 68.67, MS = 5293, *P* < 0.0001; Fig. 4(C); F(3,330) = 22.54, MS = 1812, *P* < 0.0001). AUC analysis revealed a significant reversal in weight bearing asymmetry and lowered hindpaw withdrawal thresholds over the duration of the study [Fig. 4(B), (D)].

MIA-injected rats had significant changes in the articular cartilage and synovial inflammation grades, compared to saline controls [Fig. 4(E), (F)]. Therapeutic treatment with muMab 911 did not significantly alter the MIA-induced changes in joint structure, nor the extent of synovial inflammation, compared to MIA-injected rats receiving vehicle [Fig. 4(E), (F)].

MIA-injected rats had a significant increase in the number of TRAP positive osteoclasts at the tibial plateau at 28 days, compared to saline controls [Fig. 3(B)]; numbers were comparable between the groups used for the preventative and therapeutic experiments. . At day 14 post MIA injection there is a significant (Mann–Whitney *U* = 8.500, *P* = 0.016) increase in the number of TRAP positive osteoclasts in the subchondral bone of MIA treated rats (66 ± 9, *n* = 9 rats), compared to saline treated rats (33 ± 3, *n* = 10 rats). Therapeutic muMab 911 treatment from day 14 significantly attenuated the numbers of TRAP positive osteoclasts at 28 days in the MIA rats, compared to MIA rats treated with vehicle (Fig. 3(B), (D): F (3, 34) = 7.047, MS = 2138, *P* = 0.0008).

Numbers of TRAP positive osteoclasts in the rats that had received intra-articular injection of MIA and either preventative or therapeutic treatments with muMab 911 or vehicle were positively associated with weight-bearing asymmetry (standardised beta = 0.35, *P* = 0.04), but the association with PWT did not reach statistical significance (aOR = 1.04, 95% CI = 0.997 to 1.075, *P* = 0.07).

### Effects of NGF on RANKL mediated osteoclast differentiation

**Fig. 5:**
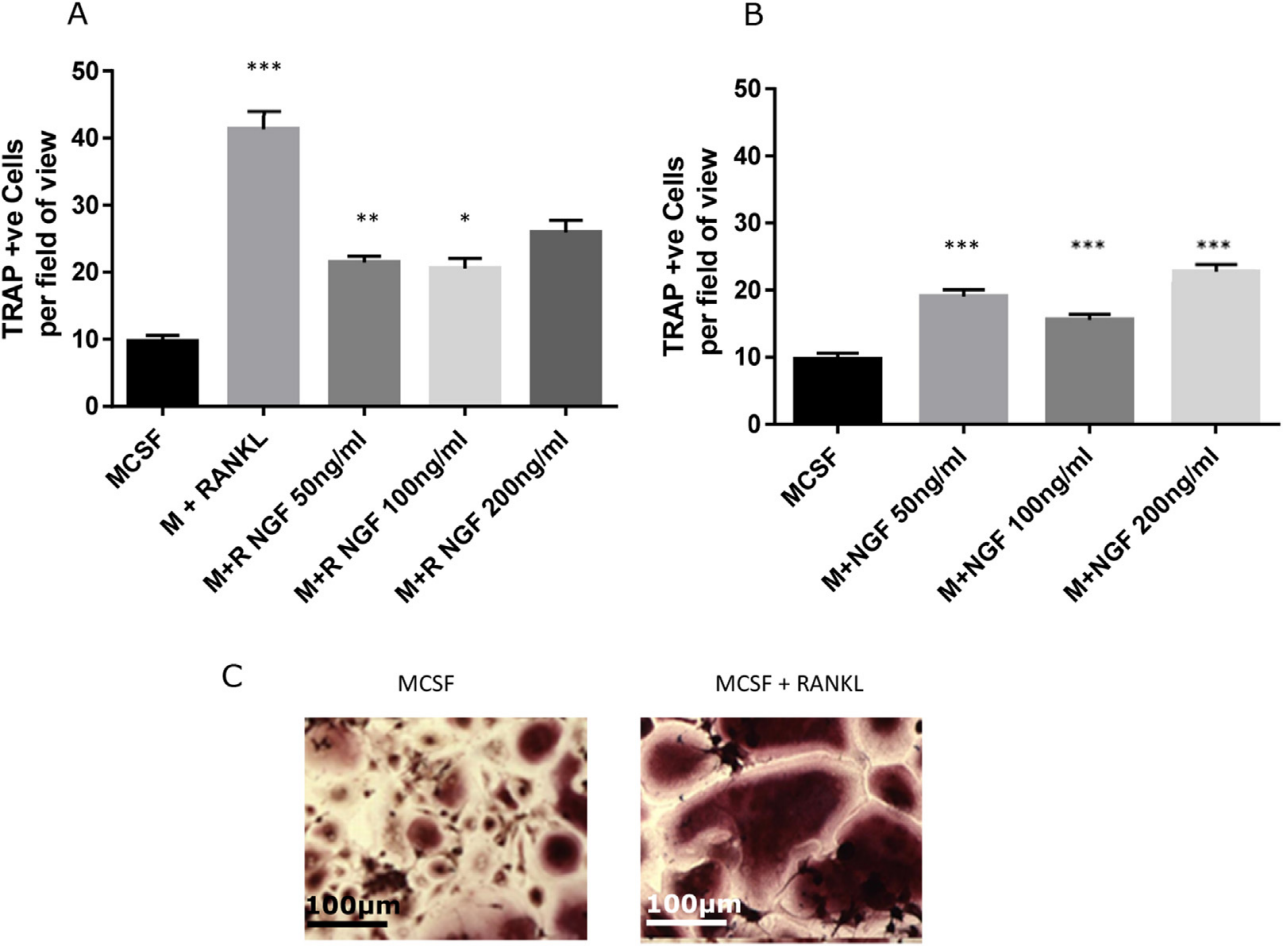
**Effects of NGF upon osteoclast differentiation *in vitro***. Human peripheral mononuclear cells were isolated and cultured in the presence of MCSF with (A) or without (B) RANKL, and increasing concentrations of NGF, for 14 days. TRAP +ve cells were counted using four random fields of vision on four separate slides for each condition. RANKL alone significantly increased TRAP +ve cells as compared to MCSF alone (A). For RANKL treated cells, addition of NGF significantly decreased osteoclast numbers at 50 and 100 ng/ml. In the absence of RANKL (B), addition of NGF caused a small non-dose related significant increase in TRAP +ve cell number. (C) Examples of TRAP staining in the presence and absence of RANKL. Scale bar is 100 μm. Data are mean ± SEM, statistical analysis: one way ANOVA, *P < 0.05, **P < 0.01, ***P < 0.001.

To determine whether the inhibitory effects of muMab 911 on numbers of subchondral osteoclasts *in vivo* is likely to arise from a direct effect of NGF on osteoclast differentiation we investigated the effect of NGF treatment on RANKL and non-RANKL mediated differentiation of human monocytes into osteoclasts. Incubation of monocytes with RANKL (30ngml^−1^) resulted in a robust increase in the number of TRAP positive multinucleated osteoclasts [Fig. 5(A)]. Lower concentrations of NGF (50 and ngml^−1^) significantly decreased the number of TRAP positive multinucleated osteoclasts, but the highest concentration of NGF (200 ngml^−1^) had no effect [Fig. 5(A)]. In the absence of RANKL, the numbers of TRAP positive multinucleated osteoclasts was markedly lower. NGF resulted in a small but significant concentration-independent increase in the number of TRAP positive multinucleated osteoclasts [Fig. 5(B)].

### Other effects of muMab 911

The appearance of expected adverse effects of muMab 911 was monitored. From Day 12 onwards of the preventative muMab 911, some rats exhibited skin irritation (Supplementary Table 1), which were treated with fuciderm gel. There were no differences in the MIA-induced pain behaviour between rats exhibiting, or not, adverse effects. One rat in the therapeutic study exhibited an adverse effect on Day 28 (Supplementary Table 1). Systemic muMab 911 treatment did not alter body weight gain compared with vehicle treatment (Supplementary fig. 3).

## Discussion

Preventative or therapeutic treatment with muMab 911 significantly prevented, or reversed, MIA-induced pain behaviour. The analgesic effects of this treatment were not associated with significant changes in chondropathy or synovial inflammation, but were associated with a significant reduction in the number of TRAP positive multinucleated osteoclasts at the tibial plateau in the model of OA. As in human OA^22^, the MIA model of OA in the rodent exhibits bone remodeling^23^, ^24^ and excessive osteoclast activity, which is significantly correlated with the magnitude of pain behavior as demonstrated previously^6^ and herein.

The analgesic effects of muMab 911 in the MIA model of OA pain are consistent with the report that the soluble NGF receptor TrkAD5, which like muMab 911 binds to NGF, blocks early and late changes in weight distribution in the DMM mouse model of OA^25^ and our recent report that blocking the TrkA receptor with a small molecule inhibitor attenuates pain behaviour in both the MIA and surgical MNX models of OA pain^26^. Control IgG1 treatment did not alter MIA-induced changes in weight bearing or PWT compared to PBS treated MIA-injected rats. The inclusion of this additional control confirms that the effects of muMAb 911 are not due to IgG1 acting as an immune modulating drug, and supports the specificity of the inhibitory effects of muMab 911 in MIA-treated rats. These preclinical data are consistent with clinical trials reporting robust analgesic effects of antibodies to NGF in OA sufferers^13^, ^14^, ^27^ and in dogs^28^. The mechanisms by which cutaneous NGF modulates sensory nerve terminal excitability, alters gene expression within the dorsal root ganglia and contributes to the development of chronic pain responses have been well studied^8^. As described earlier, although there is evidence for local changes in levels of NGF within the knee joint contributing to OA pain, the mechanisms by which these changes result in joint pain are less well characterised. The induction and release of NGF from human OA chondrocytes following both mechanical and inflammatory stimuli has been reported^29^, which may be an important source of endogenous NGF *in vivo*.

Remodeling of the subchondral bone, with initial increases in subchondral bone resorption and thinning of the subchondral plate, followed by subchondral sclerosis and osteophyte formation, is one of the structural features associated with OA^30^. The significant associations between osteoclast numbers and pain behaviour in OA rats across treatment groups presented herein is consistent with osteoclasts playing a role in OA pain. Bone is highly innervated and, unlike cutaneous innervation, the majority of bone sensory afferents express the cognate receptor for NGF, TrkA^31^. In addition, pain-related molecules have been detected in OA subchondral bone^32^.

The inhibitory effects of muMab 911 on pain behaviour were associated with a significant decrease in the numbers of TRAP positive osteoclasts within the subchondral bone, which may suggest that NGF, or factors driven by NGF, contribute to the activation of osteoclasts in OA. The inhibitory effects of muMab 911 on numbers of TRAP positive osteoclasts were evident following preventative treatment, and more importantly following therapeutic treatment which commenced at a timepoint when osteoclast number were already significantly elevated. Our data demonstrate that blockade of NGF can reverse at least one of the bone changes (increase in osteoclast number) associated with OA^21^. Osteoclast activation is associated with reduced extracellular pH and the release of proteases, and osteoclast products can activate and sensitize peripheral nerves leading to increased pain signalling^33^. Osteoclast inhibition might therefore contribute to analgesic effects of NGF blockade.

The bisphosphonate zoledronate, which targets osteoclasts, has potential analgesic, as well as structural, benefit in a subgroup of patients with OA who display bone marrow lesions on MRI^34^. Bone marrow lesions are associated with OA pain^35^, and are areas of increased metabolic activity and bone turnover, histologically characterized by subchondral bone marrow replacement by fibrovascular tissue^11^, ^23^. Although blockade of osteoclast activation and bone resorption by treatments such as the bisphosphonates inhibit pathological features of OA and the development of pain behavior both in OA models^24^ and in patients^36^, their analgesic effects are relatively weak^34^, ^36^. By contrast, anti-NGF antibody treatments have robust analgesic effects in clinical trials^14^ and as demonstrated herein in this model of OA pain. It is noteworthy that the inhibitory effects of therapeutic muMab 911 on numbers of TRAP positive subchondral osteoclasts were comparable to the inhibitory effects of therapeutic OPG-Fc treatment on osteoclast number in the same model^6^.

Neurotrophins, including NGF, are expressed in subchondral bone^11^ and by bone forming cells, and are proposed to have both autocrine and paracrine roles in bone formation^37^, ^38^. To further understand how muMab 911 inhibits the numbers of TRAP positive osteoclasts in the MIA model, we undertook an *in vitro* study to determine whether NGF can directly alter RANKL mediated differentiation of human monocytes into osteoclasts. Using the same assay for TRAP staining of multi-nucleated osteoclasts we demonstrated that a range of concentrations of NGF did not potentiate RANKL mediated osteoclastogenesis *in vitro*. Indeed, in the presence of RANKL, NGF treatment reduced the number of TRAP positive osteoclasts. In the absence of RANKL, NGF significantly increased osteoclast numbers *in vitro*, but the numbers were far smaller than when cells were treated with RANKL. Our data are consistent with a previous report that NGF has relatively minor effects on RANKL independent osteoclastogenesis *in vitro*^39^. Given levels of RANKL are known to be high within the OA knee joint, osteoclastogenesis is highly likely to occur in the presence of RANKL. The outcomes of our *in vitro* assay do not support a direct role of NGF in stimulating osteoclast function or differentiation *in vivo*.

muMab 911 treatment did not alter cartilage damage and synovial inflammation, which suggests that the effects of muMab 911 on numbers of osteoclasts within the subchondral bone were not secondary to modulation of generalised inflammatory processes within the knee joint. Consistent with other studies, our data indicate that the effects of NGF on bone turnover are complex and dependent on the underlying level and mechanisms of bone turnover. NGF blockade did not impair fracture repair in animal models^40^, but muMab 911 delayed the time to fracture in a sarcoma-induced bone cancer model^18^, suggesting that the net effect of NGF blockade may be neutral, or to increase bone strength. Collectively, evidence presented herein suggests that at least some of the subchondral bone changes associated with OA are reversible, and that inhibiting established OA pain behaviour with muMab 911 has an indirect beneficial effect on subchondral bone remodelling. Indeed, greater weight bearing in rats treated with muMab 911 may reduce osteoclastic activity and modulate bone turnover, although the anabolic effects of exercise on bone mineral density may be predominantly attributable to increased osteoblast, rather than decreased osteoclast, activity^41^.

NGF blockade using monoclonal antibodies has been associated with the rare occurrence of RPOA and further clinical trials for OA have incorporated appropriate risk mitigation strategies^42^. Our study was not designed to investigate rare adverse events. Despite the lack of significant effects of muMab 911 on joint histopathology in the current study, other effects might be restricted to discrete patient subgroups.

In conclusion, we have demonstrated beneficial effects of NGF blockade using muMab 911 in a rat model of OA pain. In addition to reducing pain behaviour, preventative and treatment protocols using muMab 911 reduced numbers of subchondral osteoclasts, indicating that this bone feature of OA is reversible and that reducing pain signalling has beneficial effects on subchondral bone changes associated with OA.

## Author contributions

All authors approved the final version of the manuscript to be published.

Professors Chapman (victoria.chapman@nottingham.ac.uk) and Walsh (david.walsh@nottingham.ac.uk) take responsibility for the integrity of the work as a whole from inception to finished article.

**Study conception and design**: Chapman, Walsh and Bennett.

**Data acquisition**: Xu, Nwosu, Sagar, Burston, Mapp, Millns, Meesawatsom, Li.

**Analysis and interpretation of data**: Xu, Nwosu, Mapp, Millns, Meesawatsom, Li, Burston, Sagar, Chapman, Walsh and Bennett.

## Conflict of interest

The authors have declared that no conflict of interest exists.

## Role of funding source

This work was funded by Arthritis Research UK, grant number 18769. LNN studentship was funded by the University of Nottingham. X.L. studentship was funded by a University of Nottingham China Scholarship.
