# Supplementary material for: The anti-NGF antibody muMab 911 both prevents and reverses pain behaviour and subchondral osteoclast numbers in a rat model of osteoarthritis pain
Source: Osteoarthritis Cartilage. 2016 Sep;24(9):1587–95. doi: 10.1016/j.joca.2016.05.015 (PMC5009895; doi:10.1016/j.joca.2016.05.015)
Supplement: Supplementary file 1 [file mmc1.docx]

**Supplementary Methods**

**Histology**

Cartilage surface integrity was scored using the OARSI cartilage histopathology assessment system [^1^](#_ENREF_1) on 3 coronal sections at 200 um intervals from the anterior half of the knee, corresponding to the region evaluated for osteoclast numbers [^2^](#_ENREF_2). Cartilage histopathology was scored from 0 (normal) to 6 (deformation) and a total joint damage score (range 0-24) was obtained by combining the cartilage score with joint involvement (0-4) [^3^](#_ENREF_3). Synovial inflammation was graded from a scale of 0 (lining layer, 1-2 cells thick) to 3 (lining layer >9 cells thick and/or severe increase in cellularity) [^3^](#_ENREF_3). Six sections from the anterior half of the knee joints per rat were prepared as previously described for tartrate-resistant acid phosphatase (TRAP) staining using a commercially available kit (F386A, Sigma-Aldrich, Dorset, UK),[^3^](#_ENREF_3).

Quantification of numbers of TRAP positive osteoclasts was perform as previously described ^[4](#_ENREF_4)^. TRAP positive osteoclasts were quantified under 40 times magnification from one end of the growth plate to the other end using the following criteria; 1) displayed purplish to dark red cytosol, 2) number of nuclei ≥3/osteoclast, 3) located within the subchondral bone area, comprising the area between the cartilage/bone junction and the growth plate. Lightly stained TRAP positive cells without nuclei identified, or located distal to the subchondral bone were not counted.

***In vitro* model of human osteoclast differentation**

Peripheral blood from healthy human donors was collected and blood monocytes were isolated from buffy coats by gradient centrifugation (Ficoll-Plague plus 1.077, GE Healthcare Bio-Sciences). Monocytes were seeded onto 13mm Ø glass coverslips within a 24-well culture plates, 500µl per well @ 3x106 cells/ml, and cultured in growth media comprising of alpha minimum essential media (αMEM) with foetal calf serum (FCS) 10%, glutamine 2mM, Penicillin 100 units/ml and Streptomycin 100µg/ml (Sigma) supplemented with 25 ng ml–1 of human macrophage colony stimulating factor (MCSF; R&D Systems) and with 30 ng ml–1 of human RANKL (Santa Cruz), unless otherwise stated. Cells were incubated at 37^o^C, 7% CO2 in a humidified incubator for 2 hours, coverslips were washed twice with αMEM without FCS so to displace unattached cells and the medium replaced. Growth media containing NGF at 0, 50, 100 or 200 ngml-1 was then added to the cells, media was changed three times a week. After 14 days, cells were washed with Hanks buffered saline solution, fixed with 10% neutral buffered formalin, washed and stored at 4oC in PBS containing 0.01% w/v sodium azide.

Differentiated osteoclasts were identified by TRAP staining using the commercial kit described above. For quantification of TRAP positive cells four random fields of view were counted per coverslip using 4 coverslips per condition. Cells that stained positive for TRAP and had 3 or more nuclei were counted.

**References**

1. Pritzker KPH, Gay S, Jimenez SA, Ostergaard K, Pelletier JP, Revell PA, et al. Osteoarthritis cartilage histopathology: grading and staging. Osteoarthritis and Cartilage 2006; 14: 13-29.

2. Gerwin N, Bendele AM, Glasson S, Carlson CS. The OARSI histopathology initiative - recommendations for histological assessments of osteoarthritis in the rat. Osteoarthritis Cartilage 2010; 18 Suppl 3: S24-34.

3. Sagar DR, Ashraf S, Xu L, Burston JJ, Menhinick MR, Poulter CL, et al. Osteoprotegerin reduces the development of pain behaviour and joint pathology in a model of osteoarthritis. Annals of the Rheumatic Diseases 2014; 73: 1558-65.

4. Parfitt AM, Drezner MK, Glorieux FH, Kanis JA, Malluche H, Meunier PJ, et al. Bone histomorphometry: standardization of nomenclature, symbols, and units. Report of the ASBMR Histomorphometry Nomenclature Committee. J Bone Miner Res 1987; 2: 595-610.

1. **Preventative muMab 911 study**

**B. Therapeutic muMab 911 study**

Day 0

7

14

21

28

OA induction

s.c. m911/PBS

**Weight-bearing asymmetry + hindpaw withdrawal thresholds**

1

4

11

18

24

Tissue collection

s.c. m911/PBS

Day 0

7

14

21

28

OA induction

s.c. PBS

s.c. PBS

s.c. m911/PBS

**Weight-bearing asymmetry + hindpaw withdrawal thresholds**

1

4

11

18

24

Tissue collection

s.c. m911/PBS

s.c. m911/PBS

s.c. m911/PBS

**Supplementary Figure 1:** Scheme of the **A)** preventative and **B)** therapeutic m911 studies. M911, muMab 911.

**Supplementary Figure 2:** **Administration of IgG1 has no significant effect on MIA-induced changes in pain behaviour**

Rats received weekly subcutaneous injection of IgG1 (10mg/kg) or PBS on days 0, 7, 14, and 21 post intra-articular injection of MIA or saline.

Preventative IgG1 did not alter MIA-induced changes in weight-bearing asymmetry (A, B) or hindpaw withdrawal thresholds (C, D). Statistical comparison of groups at each timepoint:

Two-Way ANOVA with Bonferroni’s post-hoc tests, * P<0.05, ** P<0.01, ***P<0.001: MIA-PBS vs Sal-PBS; +p<0.05, ++p<0.01, +++p<0.001 MIA-IgG vs Sal-PBS

**Supplementary Figure 3** Changes in body weight (grams) in the preventative muMab 911 study. M911.
